# The REstart or STop Antithrombotics Randomised Trial (RESTART) after stroke due to intracerebral haemorrhage: study protocol for a randomised controlled trial

**DOI:** 10.1186/s13063-018-2542-6

**Published:** 2018-03-05

**Authors:** Rustam Al-Shahi Salman, Martin S. Dennis, Gordon D. Murray, Karen Innes, Jonathan Drever, Lynn Dinsmore, Carol Williams, Philip M. White, William N. Whiteley, Peter A. G. Sandercock, Cathie L. M. Sudlow, David E. Newby, Nikola Sprigg, David J. Werring, the RESTART collaborators, Martin Dennis, Cathie Sudlow, William Whiteley, Christine Lerpiniere, Katrina McCormick, Jack Perry, Rustam Al-Shahi Salman, Ruwan Parakramawansha, Neil Hunter, Fergus Doubal, Ruth Paulton, Richard OBrien, Christine Lerpiniere, Seona Burgess, Gillian Mead, Pat Taylor, Mary-Joan MacLeod, Beverly Maclennan, Rebecca Clarke, Vicky Taylor, Katrina Klaasen, Nichola Crouch, Baljit Jagpal, Jacqueline Furnace, Janice Irvine, Heather Gow, Anu Joyson, Sandra Nelson, Sarah Ross, Ruth Davies, Deepa Jose, Naomi Robinson, Laura Codd, Angela Dodd, Helen Moroney, Pauline Weir, Victoria Little, Valerie Gott, Graeme Sangster, Paula Owings, Suja Cherian, Susan Downham, Daniel Epstein, Adam Webber, Sammie Qureshi, Paul Nicholas, Vinodh Krishnamurthy, Avani Shukla, Ifan Jones, Ashraf Ahmed, Mishell Cunningham, Tajammal Zahoor, Sharon Johnson, Carol Denniss, Mo Albazzaz, Hawraman Ramadan, Stuart Maguire, Chris Patterson, Ruth Bellfield, Brigid Hairsine, Outi Quinn, Michaela Hooley, Anand Nair, Mohammad Irfan Alam, Jill Greig, Pratap Rana, Matthew Robinson, Mahmud Sajid, Margaret Ball, Rachel Gascoyne, George Ghaly, Senthil Raghunathan, Judith Clarke, Gwendoline Wilkes, Zhe Law, Jason Appleton, Oliver Matias, Benjamin Jackson, Rekha Keshvara, Katherine Whittamore, Carla Jordan, Saima Sheikh, Jack Roffe, Nicola Gilzeane, Kailash Krishnan, Amanda Buck, Diane Havard, Amanda Hedstrom, Faye Shelton, Nikola Sprigg, Margi Godfrey, Timothy Webster, Syed Haider, Samantha Seagrave, Sandra Leason, Arumugam Nallasivan, Kausic Chatterjee, Charlotte Perkins, Azlisham Mohd Nor, Nicola Persad, Charlotte Eglinton, Claire Brown, Marie Weinling, Alex Shah, John Baker, Benjamin Hyams, Manohar Kini, Rosanna Fong, Dinesh Chadha, Deborah Walstow, Harald Proeschel, Simon Sharpe, Sarah Horton, Stephanie Jones, Anthony Byrne, Caroline McGhee, Amanda Smart, Claire Copeland, Dipankar Dutta, Rehana Bakawala, Susan O’Connell, Chloe Hughes, Pauline Brown, Fiona Davis, Kayleigh Collins, Deborah Ward, Jennifer Turfrey, Tony Rudd, Katherine Marks, Sagal Kullane, Birns Jonathan, Ajay Bhalla, Brigitte Yip, Murdina Bell, Brian MacInnes, Linda Macliver, Derek Esson, Rajendra Yadava, Samantha Stafford, Julie Reddan, Mirriam Sangombe, Khalid Azhar, Colin Jenkins, Fiona Price, Claire Hughes, Lily Mercer, Evangelos Vasileiadis, Cathy Mason, Basaam Aweid, Melinda Holden, Anthea Parry, Geraldine Landers, David Broughton, Kath Chapman, Andrew Sigsworth, Dinesh Tryambake, Andrew Young, Lynn Dixon, Adrian Bergin, Mark Barber, Derek Esson, Fiona Brodie, Talat Anjum, Lynda Connor, Susan Tucker, Sarah Thomas, Caroline Davies, Peter Slade, Shelley Treadwell, Mushtaq Wani, Teresa Beaty, Manju Krishnan, Lynne Dacey, Jayne Spencer, Leanne Quinn, Srikanth Chenna, Sharon Storton, Terry Jones, Helen Thompson Jones, Malik Hussain, Jane Homan, Eliza Foster, Lucy Brotherton, Helen Durman, Nicholas Hunt, Jayne Foot, Alison Whitcher, Corinne Pawley, Mohammad Khan, Robert Whiting, Miriam Harvey, Sarah Brown, Leanne Foote, Bella Richard, Claire Triscott, Mandy Edwards, Heidi Lawson, Rebecca Wallace, Claire Nott, Sally Moseley, Steve Buckle, Procter Sarah, Jessica Whiteman, Ken Fotherby, Donna Butler, Angela Willberry, Nasar Ahmad, Karla Jennings-Preece, Farrukh Baig, Debbie Morgan, Angela Stevens, Kneale Metcalf, Susan McDonald, Garth Ravenhill, Ajmal Anversha, Naval Shinh, Rebekah Perfitt, Richard Greenwood, Janak Saada, Kelly Waterfield, Patrick Sutton, Jenny Jagger, Alison Wiltshire, Robert Luder, Venetia Vettimootal Johnson, Hayley Bridger, Maneesh Bhargava, Jill Gallagher, Tolu Adesina, Chloe van Someren, Michael Carpenter, Marion Walker, Andrew Stanners, Julie Ball, Linda Jackson, Prabal Datta, Gavin Bateman, Razik Fathima, Richard Davey, Ann Needle, Prasad Siddegowda, Suheil Ponnambath, Anne Suttling, Yasmin Harrington-Davies, Rebecca Butler, Claire James, Stacey Valentine, Tracey Dobson, Peter Howard, Jane Tandy, Lisa Hyatt, David Jarrett, Afaq Saulat, Don Sims, Mark Willmot, Carole Green, Rachael Jones, James Cunningham, Susan Maiden, Claire Sutton, Jennifer Hurley, Edward Littleton, Raj Shekhar, Rachel Crown, Iman Ahmed, Sister Tracy Fuller, Ellie Gilham, Kelly Waterfield, Sreeman Andole, Naveen Gadapa, Karen Dunne, Magdalini Krommyda, Evelyne Burssens, Sam King, Neetish Goorah, Angela Bell, Farzana Patel, Benjamin Tomlinson, Stephen Duberley, Arun Singh, Christine Kelly, Jamie Walford, Frances Harrington, Christine Schofield, Linda Lucas, Sam Ellis, Kirsty Bond, Abhijit Mate, Katja Adie, Ali James, Bev Maund, Gillian Courtauld, Paul Mudd, Anthony Hemsley, Kevin Thorpe, Karin Gupwell, Anita Goff, Jane Sword, Caroline Roughan, David Strain, Julie Cageao, Angela Bowring, Samantha Keenan, Martin James, Hayley Kingwell, Keniesha Miller, Kirsty Harkness, Clare Doyle, Arshad Majis, Kathy Stocks, Ahmad Maatouk, Luke Barron, Katy Dakin, Ralf Lindert, Christine Kamara, Pauline Bayliss, Jessica Redgrave, Faith Kibutu, Catrin Blank, Ali Ali, Olesia Balitska, Kathryn Birchall, Emma Richards, Jo Howe, Nigel Smyth, Elio Giallombardo, Charlotte Eglinton, Lucy Sykes, Jenny Wilson, Peter Langhorne, Christine McAlpine, Louise Humphreys, Mohammad Shahzad Iqbal, Ruth Graham, Gillian Kerr, Fiona Wright, Pradeep Kumar, Philip Thomas, Charlotte Culmsee, Isabel Huggett, Linda Dunn, James Barker, Aravind Manoj, Paul Fitzsimmons, Paula Lopez, Nikhil Sharma, Penelope Cox, Glyn Fletcher, Mark Wilkinson, Hedley Emsley, Sonia Raj, Donna Doyle, Bindu Gregory, Shuja Punekar, Sulaiman Sultan, Alison McLoughlin, Kath Pasco, Olga Balazikova, Ashraf Nasim, Cassilda Peixoto, Ingrid Kane, Alexandra Pitt-Ford, Simon Hervey, Philip Thompson, Laura Latter, Emma Barbon, Joanna Breeds, Chakravarthi Rajkumar, Nicola Gainsborough, Jane Gaylard, James Choulerton, Louise Shaw, Barbara Madigan, Deborah Howcroft, Suzanne Lucas, Andrew Stone, Joanne Avis, Lukuman Gbadamoshi, Denise Button, Mc Cann Stephanie, Lindsey Dow, Michelle Davis, Teresa Thompson, Valerie Hogg, Carole Hays, Michelle Fawcett, Natalie Atkinson, Helen Guy, Stephen Woodward, Adrian Parry-Jones, Sally Marshall, Rowilson Jarapa, Stephanie Lee, Louise Harrison, Mary Johnes, Victoria Oloughlin, Edith Wood, Jane Perez, Zin Naing, Jordi Morell, Tracy Marsden, Andrea Ingham, Ilse Burger, Kelly Marie Shaw, Andrea Hall, Martin Punter, Nic Weir, Sue Evans, Ashleigh Walters, Imogen Gartrell, Fiona Smith, Chloe Cox, Charge Nurse Simon Smith, Shuna Egerton, Robyn Creeden, James Richard Marigold, Alex Blades, Pam Crawford, Lucy Sykes, Emma Battersby-Wood, Vanessa Pressly, Christopher Allen, Gabriella Howard, Keith Muir, Dheeraj Kalladka, Wilma Smith, Nicola Day, Fiona Moreton, Bharath Kumar Cheripelli, Xuya Huang, Angela Welch, Salwa El Tawil, Sankaranarayanan Ramachandran, Caroline Crosbie, Jennifer Elliot, Gillian Cluckie, Brian Clarke, Nilofer Dayal, Chukwuka Orefo, Temi Adedoyin, Rita Ghatala, Natasha Clarke, Val Jones, Adrian Blight, Caroline Lovelock, Neha Chopra, Barry Moynihan, Kate Kennedy, Rebecca Williams, Lourda Kerin, Naomi Jeyaraj, Lillian Choy, Fran Watson, Sarah Trippier, Joanna OReill, Mohammad Haque, Stuart Symonds, Mehran Maanoosi, Jane Herman, Joseph Vassallo, Shrivakumar Krishnamoorthy, Helen Cochrane, Deborah Walter, Janice OConnell, Charlotte Fox, Ramesh Krishnamurthy, Emily Osborne, Andrew Smith, Betty Mokoena, Diane Gulliver, Helen Brew, Min Myint, Nikhil Majmudar, George Bunea, Naweed Sattar, Meena Srinivasan, Indranil Mukherjee, Nichola Motherwell, Denise Donaldson, Robert Campbell, Frances Hurford, Kamy Thavanesan, Owen David, Divya Tiwari, Gail Hann, Barbara Longland, Jo Bell, Emily Rogers, Caroline Bagnall, Arshi Iqbal, Marketa Keltos, Becky Jupp, Josh Roberts, Chantel Cox, Catherine Ovington, Biju Bhaskaran, Joanne Garfield-Smith, Jean Buxton, Kathleen Horan, Afaq Saulat, Georgina Ayres, Helen Bearne, Dawn Tomlin, Susan Szabo, Debs Kelly, Isam Salih, Harbens Bhakri, Pauline Fitzell, David Wilson, Belinda Wroath, Kevin Dynan, Michael Power, Susan Thompson, Sandip Ghosh, Margo Henry, Danielle Gilmour, Elizabeth Barrie, Antony Kenton, Sheila Nyabadza, Irene Martin, Benjamin Hunt, Hardi Hassan, Bander Dallol, Girish Muddegowda, Joanne Hiden, Holly Maguire, Jeanette Grocott, Kay Finney, Adrian Barry, Christine Roffe, Sue Lyjko, Ranjan Sanyal, Alda Remegoso, Phillip Ferdinand, Adrian Butler, Nenette Abano, Chelsea Causley, Hayley Denic, Racquel Carpio, Stephanie Stevens, Andrew Moores, Resti Varquez, Yogish Pai, David Bruce, Sofia Dima, Vidya Baliga, Muhammad Naeem, Gill Rogers, Ellen Brown, Rachel Hayman, Mark Garside, Mahesh Dhakal, Gemma Marie Smith, Susan Clayton, Enoch Orugun, Una Poultney, Rachel Glover, Hannah Crowther, Sarah Thornthwaite, Tom Webb, Eva Beranova, Susannah Walker, Tracey Cosier, Hannah Rudenko, Linda Cowie, Anna Verrion, Audrey Thomson, Ed Gamble, Bethan Charles, Rebecca Grue, Sujata Blane, Adam Hague, Khalid Rashed, Carinna Vickers, Diane Wood, Janne Board, Clare Buckley, Joanna Allison, Sarah Board, Barbara William-Yesson, Linda Balian, Elizabeth Keeling, Arindam Kar, Omid Halse, Vinh Nguyen, Kirsten Harvey, Léjeune Gardener, Sheila Mashate, Victoria Tilley, Peter Wilding, Olivia Geraghty, Beth Hazel, Thomas Harrison, Larissa Cuenoud, Grace Auld, Esther Erumere, Ozlem Redjep, Gemma Grimwood, Laura Howaniec, Dionne Hove, Afraim Salek-Haddadi, Kari Saastamoinen, Lucia Argandona, Ivan Wiggam, Aine Wallace, Sarah Cuddy, Suzanne Tauro, Annemarie Hunter, Enda Kerr, Ailsa Fulton, Janet Putterill, Puneet Kakar, Ratneshwari Jha, Rachel Gallifent, Aparna Pusalkar, Kelly Chan, Puneet Dangri, Karen Crabtree, Hannah Beadle, Angela Cook, Toby Black, Julie Cronin, Ruth Fennelly, Michele Tribbeck, Caroline Clarke, Skelton Miriam, Alpha Anthony, Denise Mead, Bernard Esisi, Maria Bokhari, Tim Cassidy, Beverley McClelland, Martin Cooper, Inez Wynter, Anoja Rajapakse, Mohammad Nasar, Ijaz Anwar, Lynn Dixon, Alex Ramshaw, Arunkumar Annamalai, Susan Crawford, Tarn Nozedar, Helen Skinner, Balakrishna Kumar, Damian McArdle, Clare Holmes, Emily Dodd, Samantha Clarke, Sarah Caine, Pauline Baker, Peter Murphy, Mairead Osborn, Lucy Belle Guthrie, Amy Steele, Nicola Devitt, David Mangion, Jo Fletecher, Anne Hardwick, Carmen Constantin, Skarlet Markova, Tara Lawrence, Santhosh Subramonian, Natalie Temple, Peter Owusu-Agyei, Nicola Butterworth-Cowin, David Werring, Caroline Hogan, Maria Brezitski, Emma Elliott, Nina Francia, Amy Ashton, Isabel Hostettler, Nnebuife Oji, Azra Banaras, Krishna Patel, Luci Crook, Caroline Watchurst, Duncan Wilson, Ifan Jones, Renuka Erande, Lakshmanan Sekaran, Niaz Mohammed, Meena Chauhan, Sakthivel Sethuraman, Rohan Simon, Kiranjit Bharaj, Margaret Tate, Frances Justin, Duke Phiri, Jonathan Hewitt, Claire Nott, Jane Gray, Rebecca Wallace, Rina Mardania, Claire Triscott, Sarah Procter, Steve Buckle, Jessica Whiteman, Khaled Elfandi, Julie Reddan, Uzma Khan, Samantha Stafford, Suzanne Ragab, Kerstin Knops, Emma Jinks, Christine Dickson, Laura Gleave, Jacqui Leggett, Judith Dube, Tatiana Garcia, James McIlmoyle, Sajjad Anwar, Saikat Dhar, Kirsty Jones, Carol Jeffs, Christina Dickinson, Joanne Howard, Timothy England, Richard Donnelly, Amanda Hedstrom, Mohana Maddula, Ahamad Hassan, Emelda Veraque, Mary Kambafwile, Linetty Makawa, Marc Randall, Vasileios Papavasileiou, Dean Waugh, Sissy Ispoglou, Anne Hayes, Sandeep Ankolekar, Rachel Evans, Hlaing Ni, Carol Graham, Josin Jose, Josette Milligan, Bithi Rahman, Paul Findlay, Ashish Macaden, Ian Shread, Breffni Keegan, Caroline Blair, Jim Kelly, Mandy Doherty, Richard Dewar, James White, Kelly Thomas, David Cohen, Anette David, Emmanuelle Owoyele, Kelechi Njoku, Philip Poku, Varthi Sukdeo, Aberami Chandrakumar, Angela Chamberlain, Mudhar Abbdul-saheb, Fenglin Guo, Anne Oshodi, Radim Licenik, Joseph Devine, Silvie Davies, Nabeela Nisar, Rangah Niranchanan, Tatjana Roganova, Mushiya Mpelembue, Laura Burgess, Rajaram Bathula, Mmua Ngwako, David Eveson, Amit Mistri, Claire Stephens, Kashif Musarrat, Man Yee Lam, Saira Sattar, Shagufta Khan, Mohammed Moqsith, Lisa Manning, Champa Patel, Ursula Schulz, James Kennedy, Gary Ford, George Harston, Rachel Teal, Philip Mathieson, Giulia Lenti, Ian Reckless, Claire Cullen, Sarah Stevenson, Melanie Harrison, Jordan Ewing, Daniela Shackcloth, Ramesh Durairaj, Mellor Zoe, Tanya Ingram, Hlaing Thant, Jenny Peters, Victoria Sutton, Simone Ivatts, Isobel Amey, Lisa Clayton-Evans, Yolanda Baird, Moore Sally, Sophie Newton, Paul Guyler, Kheng Xiong Ng, Rajalakshmi Orath Prabakaran, David Ngo, Sindhu Rashmi, Lucy Coward, Nisha Menon, Shyam Kelavkar, Swapna Kunhunny, Devesh Sinha, Anwer Siddiqui, Amber Siddiqui, Thayalini Loganathan, Sharon Tysoe, Sweni Shah, Latheef Kalathil, Nikki Gautam, Julie Meir, Duncan Bailey, Maqsud Salehin, Richard Miller, Amor Kelly, Rayessa Rayessa, Alicia Rodgers, Lisa Wilson, Charde Naylor, Sarah Wilson, Emma Clarkson, Mark McCarron, Ferghal McVerry, Caroline Blair, Jacqueline McKee, Mandy Doherty, Vera Cvoro, Khalil Ullah, Nicola Chapman, Mandy Couser, Katrina McCormick, Sean Mcauley, Susan Pound, Anne Nicolson, Javed Imam, Julie White, Lisa Wood, Eoin OBrien, Niamh Hannon, Sarah Finlay, Helen Hayhoe, Dominic Handley, Siobhan Kelly, Joanne Mcgee, Jennifer Mitchell, Elaine Amis, Juliana Sesay, Sarah Crisp, Jobbin Francis

**Affiliations:** 1 Centre for Clinical Brain Sciences, University of Edinburgh, Chancellor’s Building, 49 Little France Crescent, Edinburgh, EH16 4SB UK; 2 Usher Institute of Population Health Sciences and Informatics, University of Edinburgh, Edinburgh, UK; 3 Institute of Neuroscience and Newcastle University Institute for Ageing, Newcastle-upon-Tyne, UK; 4 Centre for Cardiovascular Science, University of Edinburgh, Edinburgh, UK; 5 Faculty of Medicine and Health Sciences, University of Nottingham, Nottingham, UK; 6 Institute of Neurology, University College London, London, UK

**Keywords:** Secondary prevention, Antiplatelet therapy, Stroke, Intracerebral haemorrhage, Randomised controlled trial

## Abstract

**Background:**

For adults surviving stroke due to spontaneous (non-traumatic) intracerebral haemorrhage (ICH) who had taken an antithrombotic (i.e. anticoagulant or antiplatelet) drug for the prevention of vaso-occlusive disease before the ICH, it is unclear whether starting antiplatelet drugs results in an increase in the risk of recurrent ICH or a beneficial net reduction of all serious vascular events compared to avoiding antiplatelet drugs.

**Methods/design:**

The REstart or STop Antithrombotics Randomised Trial (RESTART) is an investigator-led, randomised, open, assessor-blind, parallel-group, randomised trial comparing starting versus avoiding antiplatelet drugs for adults surviving antithrombotic-associated ICH at 122 hospital sites in the United Kingdom. RESTART uses a central, web-based randomisation system using a minimisation algorithm, with 1:1 treatment allocation to which central research staff are masked. Central follow-up includes annual postal or telephone questionnaires to participants and their general (family) practitioners, with local provision of information about adverse events and outcome events. The primary outcome is recurrent symptomatic ICH. The secondary outcomes are: symptomatic haemorrhagic events; symptomatic vaso-occlusive events; symptomatic stroke of uncertain type; other fatal events; modified Rankin Scale score; adherence to antiplatelet drug(s). The magnetic resonance imaging (MRI) sub-study involves the conduct of brain MRI according to a standardised imaging protocol before randomisation to investigate heterogeneity of treatment effect according to the presence of brain microbleeds. Recruitment began on 22 May 2013. The target sample size is at least 720 participants in the main trial (at least 550 in the MRI sub-study).

**Discussion:**

Final results of RESTART will be analysed and disseminated in 2019.

**Trial registration:**

ISRCTN71907627 (www.isrctn.com/ISRCTN71907627). Prospectively registered on 25 April 2013.

**Electronic supplementary material:**

The online version of this article (10.1186/s13063-018-2542-6) contains supplementary material, which is available to authorized users.

## Background

Patients with stroke due to spontaneous intracerebral haemorrhage (ICH) often have past histories of systemic arterial hypertension, smoking, and diabetes mellitus. These risk factors also contribute to the occurrence of other conditions before ICH, such as ischaemic stroke, coronary artery disease, and atrial fibrillation. These risk factors and diseases may also cause vaso-occlusive events after ICH, which – overall – appear to occur with a similar frequency to recurrent ICH [1].

Among individuals at high risk because of a prior vaso-occlusive event, aspirin provides statistically and clinically significant absolute reductions in all serious vascular events from 8.2% to 6.7% per year, in all stroke from 2.5% to 2.1% per year, and in coronary events from 5.3% to 4.3% per year, despite a non-significant increase in the risk of intracranial haemorrhage [2]. Antiplatelet drugs also seem to be beneficial for preventing vaso-occlusive events in patients with atrial fibrillation and no past history of vaso-occlusive events, without a detectable increase in the risk of extracranial or intracranial haemorrhage [3]. However, patients with spontaneous ICH were not included in the randomised controlled trials (RCTs) contributing to these analyses [2, 3], but it is likely that the benefits of secondary prevention with antiplatelet drugs would apply after ICH (although whether they are outweighed by the risk of recurrent ICH is unknown).

There are no published RCTs comparing the effects of starting versus avoiding antiplatelet drugs for the prevention of vaso-occlusive disease in adults after ICH [4]. Four small, non-randomised observational studies have not identified consistently beneficial or harmful outcomes associated with starting or avoiding antiplatelet drugs after ICH [5–8]: one study found that aspirin was associated with a beneficial reduction in the composite endpoint of recurrent ICH, ischaemic stroke or acute coronary syndrome during follow-up in the sub-group of patients with ICH and indications for aspirin [6], whilst another study of 104 survivors of lobar ICH found an increase in the subsequent risk of recurrent ICH associated with aspirin in multivariable analyses [5].

Because the benefits of antiplatelet drugs for the prevention of vaso-occlusive disease are likely to continue to apply after ICH and because the effect of antiplatelet drugs on the risk of recurrent ICH is unknown, it is reasonable to consider starting antiplatelet drugs. However, because the published observational studies have not provided evidence of a substantial increase in the risk of recurrent ICH with antiplatelet drugs in such patients, a RCT is needed to assess the balance of benefits and harms. A similarly designed RCT has proven feasible and acceptable for patients who had been taking low-dose aspirin before peptic ulcer bleeding [9].

Therefore, our aim is to conduct a RCT to determine whether antiplatelet drugs increase the risk of recurrent symptomatic ICH to an extent that might outweigh any beneficial reduction in vaso-occlusive disease. The results of this RCT may indicate whether a subsequent large-scale RCT is needed to determine whether the gains from prevention of vaso-occlusive disease with antiplatelet drugs outweigh the risks of intracranial and extracranial haemorrhage, and hence whether there is net benefit for patients after ICH associated with antithrombotic drug use. Here, we report the final version of the trial protocol, compliant with the SPIRIT reporting guideline (Additional file 1).

## Methods/design

### Study setting

At the time of writing, RESTART collaborators are based at 122 hospital sites in the National Health Service in the United Kingdom (see the ‘Acknowledgements’ section below for a list of these sites and collaborators). In view of slow recruitment [10], we conducted the Promoting Recruitment using Information Management Efficiently (PRIME) stepped-wedge, cluster randomised study within a trial (SWAT) at 72 sites to determine whether a complex intervention could boost recruitment [11, 12]. One hundred and four sites are recruiting participants to the sub-study, which involves the conduct of brain magnetic resonance imaging (MRI) before randomisation according to the MRI sequences and parameters specified by an imaging protocol (www.restarttrial.org/documents/RESTART_MRI_protocol.pdf).

### Eligibility criteria

Inclusion criteria: patient aged 18 years or older; spontaneous ICH not attributable to preceding head injury and either ‘secondary’ to an underlying structural cause (e.g. aneurysm, tumour, arteriovenous malformation, or intracranial venous thrombosis), or ‘primary’ (if the investigator either does not suspect an underlying structural cause, or it is not detected by further radiographic investigation); patient had taken an antithrombotic (i.e. anticoagulant or antiplatelet) drug for the prevention of vaso-occlusive disease for any length of time before the onset of the qualifying ICH; patient is at least 24 h after ICH symptom onset; patient and their physician are both uncertain about whether to start or avoid antiplatelet drugs; patient is registered with a general (family) practitioner (GP); brain imaging study that first diagnosed the qualifying ICH is available; consent to randomisation; if eligible for the brain MRI sub-study, the MRI must be performed after the ICH but before randomisation. Exclusion criteria: ICH due to head injury or haemorrhagic transformation of an ischaemic stroke, in the opinion of the investigator; patient is taking an anticoagulant drug following ICH; patient is pregnant, breastfeeding, or of childbearing age and not taking contraception; patient and carer unable to understand spoken or written English (local translator is not available); patients are ineligible for the brain MRI sub-study if they are claustrophobic or they have a contraindication to MRI.

### Interventions

RESTART randomises participants to policies of starting or avoiding antiplatelet drugs. The intervention is restricted to the use of one or more of aspirin, dipyridamole (to which prior ICH is not a contraindication in the Summary of Product Characteristics (SPC)) or clopidogrel (to which only ‘active pathological bleeding’ (judged to occur within the first 24 h of ICH symptom onset) is a contraindication). The specific antiplatelet drug(s) and their doses are determined at the discretion of the consultant responsible for the participant and should be prescribed to start within 24 h of randomisation. The comparator is a policy of avoiding antiplatelet drugs; there is no placebo. Participants may discontinue antiplatelet drugs (e.g. if a bleeding outcome event occurs) or if the prescription of a contraindicated medication is required in a participant taking antiplatelet drugs (e.g. if a vaso-occlusive outcome event occurs which requires treatment with an anticoagulant). To increase the likelihood that participants will receive antiplatelet drugs if allocated to a policy of starting them, RESTART: encourages the randomising clinician to emphasise the importance of adhering to the allocated treatment policy; writes to the GP shortly after enrolment to alert them to the participant’s inclusion and treatment allocation in the trial; writes to the participant at home (when the hospital/clinic discharge form is received) reminding them of the purpose of RESTART and the importance of adhering to their treatment allocation. RESTART monitors adherence in all participants regardless of treatment allocation by recording antiplatelet and anticoagulant use: after randomisation on the discharge form; on annual participant questionnaires; on annual GP questionnaires; and from *ad hoc* reports by participants or their carers to the trial coordinating centre.

### Outcomes

RESTART uses multiple sources of ascertainment to detect outcome events, including: the hospital/clinic discharge form after randomisation; annual participant questionnaires; annual GP questionnaires; ad hoc reports from participants, carers, and GPs.

Primary outcome:

- Fatal or non-fatal radiographically or pathologically proven, recurrent, symptomatic ICH (defined as the abrupt onset of headache, altered level of consciousness, or focal neurological deficit, anatomically referable to a focal collection of blood predominantly located within the brain parenchyma (diagnosed on brain imaging or at autopsy), which was not attributable to prior trauma or haemorrhagic transformation of an ischaemic stroke)

Secondary outcomes:

- Fatal (i.e. followed by death within 30 days) or non-fatal (i.e. not followed by death within 30 days) serious vascular events:
  - ○ Symptomatic haemorrhagic events
    - ▪ Symptomatic spontaneous or traumatic extradural haemorrhage, subdural haemorrhage, subarachnoid haemorrhage, or intraventricular haemorrhage (not accompanying spontaneous ICH)
    - ▪ Symptomatic major extracranial haemorrhage, sub-divided by site (requiring transfusion or endoscopic treatment or surgery, or resulting in death within 30 days)
  - ○ Symptomatic vaso-occlusive events
    - ▪ transient ischaemic attack
    - ▪ ischaemic stroke
    - ▪ acute coronary syndrome
    - ▪ peripheral arterial occlusion
    - ▪ mesenteric ischaemia
    - ▪ retinal arterial occlusion
    - ▪ deep vein thrombosis
    - ▪ pulmonary embolism
    - ▪ revascularisation procedures (carotid, coronary, or peripheral arterial)
    - ▪ cardiac death with symptoms suggestive of myocardial ischaemia (type 3), or evidence of arrhythmia
  - ○ Symptomatic stroke of uncertain sub-type
    - ▪ Non-fatal stroke, with brain imaging performed too late to distinguish ICH from cerebral infarction
    - ▪ Rapidly fatal stroke, but without radiographic or pathological confirmation
- Other fatal events
- Annual ratings of participant function completed by participant or their carer:
  - ○ Simplified modified Rankin Scale postal questionnaire [13, 14]
  - ○ Structured telephone interview with non-responders to the postal questionnaire [15]

### Participant timeline

**Fig. 1:**
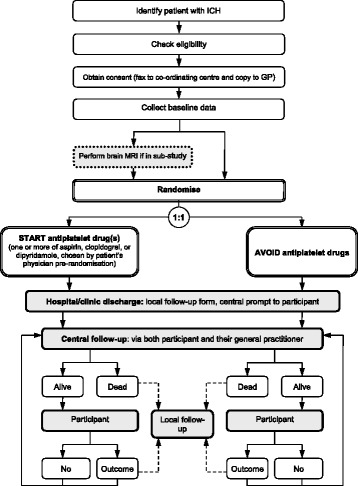
A flowchart describing the REstart or STop Antithrombotics Randomised Trial (RESTART) trial design

**Fig. 2:**
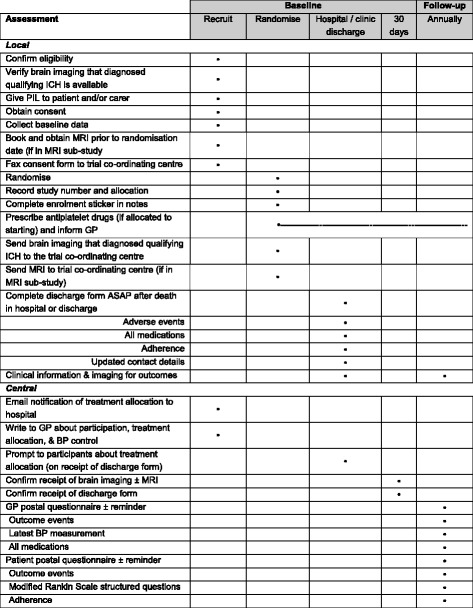
Schedule of enrolment, interventions, and assessments

Figures 1 and 2 illustrate the schedule of enrolment, randomisation, treatment allocation, and assessments for participants.

### Co-enrolment

Inclusion in another research study, including another RCT but not including a phase I or first-time-into-human study, does not preclude participation in RESTART as long as: participants are not overburdened; their inclusion would not confound RESTART’s results or complicate attribution of serious adverse events and outcomes; and co-enrolment has been agreed with the chief investigators of all studies involved in co-enrolment. Participants in TICH2 (www.tich-2.org, ISRCTN93732214) may be co-enrolled in RESTART if at least 21 days have elapsed after enrolment in TICH2 and the terms of the co-enrolment agreement are upheld [16].

### Sample size

There is considerable uncertainty about the absolute risks of recurrent symptomatic ICH among survivors who were taking antiplatelet drugs at the time their index event, but a review of the academic literature suggests that the event rate lies in the range of about 1.8 to 7.4% per year [1]. Similarly, information about the relative increase in the risk of recurrent ICH on antiplatelet drugs is scarce, but estimated relative risks from non-randomised studies have ranged from no excess (relative risk (RR) = 1) to a four-fold excess (RR = 4) [5–7]. This study of 720 patients will have excellent power (after all participants have been followed for at least 2 years) to detect a doubling of the rate of ICH if the true rate is 4.5% per annum [2, 5, 7, 17], but there would be 93% power at the 5% significance level to detect a four-fold increase in risk of recurrent ICH if the annual risk is only 1%. In both these scenarios the absolute excess risk of recurrent ICH may be higher than any plausible benefit of treatment, in which case it may be inappropriate to consider a larger trial designed to demonstrate net benefit. The Trial Steering Committee will review the target sample size and adjust this based on accruing data on: the number of primary outcome events, completeness of follow-up, and the enrolment into specific pre-specified sub-groups (e.g. lobar ICH location). Contraindications to MRI, claustrophobia, non-attendances, and scheduling constraints preclude MRI in approximately 25% of patients, so we hope to obtain brain MRI on approximately 550 patients before randomisation.

### Recruitment and retention

Potentially eligible patients are identified in the everyday clinical practice of research staff, or referred to them for assessment of eligibility having been identified elsewhere (e.g. by clinical staff who are not research staff in secondary or primary care). Collaborators screen new admissions to stroke services and may identify patients looked after by their stroke service for ICH in the past using data extracts from stroke audits that were developed for the PRIME SWAT (e.g. https://www.strokeaudit.org/Research/Data-requests/Accepted-aggregate-data-requests.aspx) [11], or their stroke service’s database. Collaborators identify patients at least 24 h after ICH symptom onset and at a time when uncertainty arises about whether to avoid or start antiplatelet drugs. There is no specific time window for identifying participants, so they may be recruited during their hospital admission for the qualifying ICH or at a later stage in an outpatient clinic. The principal investigator or physician with delegated responsibility is responsible for confirming eligibility, ensuring informed consent is obtained and that the informed consent form is completed, signed and dated by all parties and faxed to the RESTART trial office before randomisation. Local research staff follow the laws that govern consent procedures in their jurisdiction, in particular those governing incapacitated adults: if a patient lacks capacity to consent for themselves then a personal/legal/professional representative may provide consent on the patient’s behalf. Consent must also be provided explicitly if the participant is to undergo brain MRI specifically for RESTART. Annual follow-up is coordinated by the trial coordinating centre, and begins with a postal questionnaire to each participant’s GP (followed by a telephone reminder if required) to establish each participant’s vital status, the occurrence of primary and secondary outcome events, hospital admissions, a recent blood pressure (BP) measurement, a list of current medications (specifically enquiring about antithrombotic and antihypertensive drugs), and up-to-date contact details for the participant. If the participant is still alive and appropriate for questionnaire follow-up, the participant will be sent a postal questionnaire (followed by a telephone reminder if required) to establish the occurrence of primary and secondary outcome events, hospital admissions, antiplatelet drug use, and their current modified Rankin Scale score [13–15]. If the participant does not have capacity or cannot speak English, their carer will be asked to complete and return the forms. If the follow-up information cannot be obtained by either postal or telephone questionnaire the local research team is asked to assist. The chief investigator (RA-SS) makes the final attempt to obtain missing GP/participant follow-up. A telephone helpline is available for participants, carers or GPs to report or discuss outcome events. These methods have been reviewed and approved by the patient reference group for the Research to Understand Stroke due to Haemorrhage (RUSH) programme (www.RUSH.ed.ac.uk).

### Allocation

Having obtained consent, the researcher collects the baseline data necessary to complete a randomisation form, enters the participant’s baseline data into a computerised central randomisation service by means of a secure round-the-clock web interface or a telephone call to the trial office during office hours (if the web interface is not operational). The web interface checks these baseline data for completeness and consistency. To avoid predictable alternation of treatment allocation (and potential consequent loss of allocation concealment) a minimisation algorithm randomly allocated the first participant with a probability of 0.5 to one arm of the trial, but the randomisation algorithm for each subsequent participant involves adaptive stratification (i.e. minimisation) and allocates them with a probability of 0.8 to the group which minimises differences between the two arms of the trial with respect to five variables collected by research staff at baseline. The participant is then allocated a unique study identification number and their allocation to starting or avoiding antiplatelet drugs is displayed on the web interface and in an email to all local research staff at the hospital site, having been concealed until that point. If the participant is allocated to starting antiplatelet drugs, they are reminded about the antiplatelet drug that would be prescribed as stated on the randomisation form.

### Masking (blinding)

Treatment allocation in RESTART is not masked, and therefore it is open to participants, the clinicians caring for them in secondary and primary care, and local research staff. Central research staff carrying out follow-up are masked to participants’ treatment allocation when obtaining information (questionnaires and the trial database do not reveal treatment allocation to them), as are outcome event adjudicators (they review source documentation of events that might be outcomes with identifiable and treatment information redacted).

### Data collection methods

Research staff at collaborating hospital sites collect all baseline data on demographics, co-morbidities, the qualifying ICH, and treatments from participants or their medical records. The patient’s hospital consultant indicates the intended antiplatelet therapy if randomised to starting antiplatelet drugs, which is collected before randomisation. Validation rules embedded within the web interface ensure 100% completeness of baseline data. Images that diagnosed the qualifying ICH and any brain MRI sub-study images that were obtained before randomisation are copied in Digital Imaging and Communications in Medicine (DICOM) format, pseudonymised with the participant ID, and sent to the trial coordinating centre after randomisation. Following receipt, the RESTART imaging manager checks each imaging study to ensure that it relates to the appropriate participant at the appropriate time, that it is the appropriate modality, and that all the required images and sequences have been sent; after quality assurance these images are uploaded to an electronic archive and allocated to one of a panel of consultant neuroradiologists via the in-house, web-based, systematic image review system (https://www.ed.ac.uk/clinical-sciences/edinburgh-imaging/research/imaging-services-for-research/trial-images-service-smartis) for confirmation of ICH diagnosis, ICH location, and other radiographic evidence of small-vessel disease [18–20]. Outcome data are collected using structured postal/telephone questionnaires (available from the corresponding author upon reasonable request). Completeness of follow-up information is maximised by preventing the inclusion of participants who do not have a GP, monitoring completeness of follow-up at fortnightly team meetings, limiting the collection of unnecessary data from investigators and participants, emphasising retention of participants until the end of the trial, and ensuring that participants’ contact details are kept up to date. There are no provisions for ancillary and post-trial care given that the intervention being tested is available in standard clinical practice.

### Data management

All records are kept in a secure storage area with limited access, clinical information will not be released without the written permission of the participant, and research staff may not disclose or use for any purpose other than performance of the study, any data, record, or other unpublished, confidential information disclosed to them for the purpose of the study. All research staff must comply with the requirements of the Data Protection Act 1998 with regard to the collection, storage, processing and disclosure of personal information and uphold the Act’s core principles. Data completeness, range, and consistency checks are performed at randomisation and hospital/clinic discharge, as well as on imaging data collection forms. Incomplete data on follow-up forms are chased by the chief investigator. Documentation about RESTART’s data management procedures can be obtained from the corresponding author upon reasonable request.

### Statistical methods

We intend to publish a final Statistical Analysis Plan as an update to this protocol before the database is locked for analysis and the results are known. In order to preserve fully the huge benefit of randomisation, we will include all randomised participants in the analysis (irrespective of whether they adhere to the allocated treatment), all retained in the group to which they were allocated (i.e. ‘as-randomised’). This will comprise a Kaplan-Meier survival analysis of time to first outcome event after randomisation. Follow-up will be censored at death (unrelated to an outcome event), last available follow-up, or voluntary withdrawal from the trial. We will compare the survival function in the two trial arms using a Cox proportional hazards regression model, adjusting for all the covariates included in the minimisation algorithm, and presenting the result as an estimated adjusted hazard ratio with its corresponding 95% CI. We will also report the unadjusted estimate of the hazard ratio and its corresponding 95% CI, together with the result of the log-rank test. We will use available data and not impute missing data. We will analyse heterogeneity of treatment effect using statistical tests of interaction.

### Data monitoring

A Data Monitoring Committee (DMC), which is independent of the sponsor, oversees the safety of participants in the trial, according to the terms of reference in the DMC charter, which is available from the corresponding author on reasonable request. During the period of recruitment into the study, interim analyses of the baseline and follow-up data are supplied, in strict confidence, to the chairman of the DMC, along with any other analyses that the committee may request. In the light of these analyses, the DMC will advise the independent chair of the Trial Steering Committee if, in their view, the randomised comparisons have provided both (1) ‘proof beyond reasonable doubt’ that for all, or some, the treatment is clearly indicated or clearly contra-indicated and (2) evidence that might reasonably be expected to materially influence future patient management. Appropriate criteria of proof beyond reasonable doubt cannot be specified precisely, but the DMC will work on the principle that a difference of at least three standard errors in an interim analysis of a major outcome event (e.g. the primary outcome) may be needed to justify halting, or modifying, a trial before the planned completed recruitment. This criterion has the practical advantage that the exact number of interim analyses would be of little importance, and so no fixed schedule is proposed. Unless this happens, however, the Trial Steering Committee, the collaborators and central administrative staff will remain ignorant of the interim results.

### Harms

RESTART is a pragmatic RCT involving antiplatelet drugs which have well-established safety profiles. The trial will routinely collect data on outcomes, serious adverse events (SAEs), and serious unexpected adverse reactions (SUSARs), and these will be reviewed by the independent DMC. The trial procedures are based on routine clinical procedures and include (1) prescription of antiplatelet drugs in routine clinical practice for standard indications, (2) collecting routine clinical information from the medical records, and (3) informed consent. There are no complex procedures or interventions for the participants or research staff in this trial. Clinical management for underlying conditions will remain as per each hospital’s standard protocol. Based on these factors, the probability of harm or injury (physical, psychological, social or economic) occurring as a result of participation in this research study is considered to be low in each of these categories. The university has insurance in place (which includes no-fault compensation) for negligent harm caused by poor protocol design by employees of Edinburgh University. Sites participating in the study will be liable for clinical negligence and other negligent harm to individuals taking part in the study and covered by the duty of care owed to them by the sites concerned. Sites which are part of the United Kingdom’s Nation Health Service will have the benefit of NHS Indemnity.

### Auditing

The RESTART internal monitoring procedure to assure appropriate conduct of the trial uses a combination of central data monitoring and remote self-monitoring unless issues are identified that can only be addressed by site monitoring in accordance with the Monitoring Plan agreed by the sponsor. This is regularly reviewed during the course of the trial.

### Dissemination policy

This report of the protocol complies with the Standard Protocol Items: Recommendations for |Interventional Trials (SPIRIT) 2013 reporting guideline [21, 22]. On completion of the trial, the data will be analysed and tabulated, a clinical study report will be prepared in accordance with Good Clinical Practice (GCP) guidelines, and a manuscript for publication will be prepared in accordance with Consolidated Standards of Reporting Trials (CONSORT) guidelines. Active collaborators included in the delegation logs at sites that have recruited participants will be included in any listing of collaborators. The primary trial publication will be drafted by a writing committee whose membership has been approved by the Trial Steering Committee, who will approve the manuscript before submission for publication. Results will be disseminated to participants who have opted in to receive them at the time of providing informed consent, and via social media channels (https://twitter.com/BleedingStroke and https://www.facebook.com/bleedingstroke).

### Data sharing

Ownership of the data arising from this study resides with the Trial Steering Committee. Consistent with recent guidance [23], access to the datasets generated and/or analysed during RESTART will be available on reasonable request after the publication of the main results. Access will be controlled by the chief investigator, with the approval of the Trial Steering Committee. Trial participants or their proxies are provided with assurances about the maintenance of privacy and confidentiality in the information leaflet and they are asked to consent to the statement, ‘I agree that information about me and my brain scans may be used in other ethically approved research studies in the future as long as all the information (including the images) is anonymised’.

## Discussion

Since RESTART began in 2013, recruitment has been challenging. We have learned that one in 12 eligible patients is actually randomised, mostly because morbidity and mortality after ICH and physician certainty are impediments to recruitment [10]. We have tried to boost recruitment with a variety of promotional activities, including: patient-orientated information on our website (http://www.restarttrial.org/patient.html), monthly collaborator newsletters, annual collaborator meetings, a complex recruitment intervention which has been rolled out to all sites after the PRIME SWAT was completed [11], and one-to-one engagement with site investigators by the chief investigator by telephone and in person.

Other similar RCTs are underway in France (https://clinicaltrials.gov/ct2/show/NCT02966119) and Scandinavia (https://www.clinicaltrialsregister.eu/ctr-search/trial/2014-002636-13/SE) [4]. The chief investigators of these studies have formed a Collaboration Of Controlled Randomised trials of Oral Antithrombotic drugs after intraCranial Haemorrhage (COCROACH), with the intent of performing a prospectively planned, individual-patient, data meta-analysis of these RCTs to maximise power to detect the effects of antithrombotic drugs in these patients overall, and in sub-groups of interest (www.ed.ac.uk/clinical-brain-sciences/research/so-start/for-collaborators).

## Trial status

The first participant was randomised on 22 May 2013. Recruitment is on-going and will end on 31 May 2018. The current protocol is version 7.0, created on 23 December 2015 (all protocol updates since version 3.0, created 1 February 2013, which was implemented before randomisation began, have been approved by the sponsor and Research Ethics Committee and also communicated to investigators and trial registries).

## Additional file

Additional file 1: RESTART protocol SPIRIT Checklist. (DOCX 50 kb)

## Abbreviations

DMC: Data Monitoring Committee
GP: General (family) practitioner
ICH: Intracerebral haemorrhage
MRI: Magnetic resonance imaging
PRIME: Promoting Recruitment using Information Management Efficiently
RCT: Randomised controlled trial
RESTART: REstart or STop Antithrombotics Randomised Trial
SWAT: Study within a trial
